# Aquatic macroinvertebrates in Uruguayan rice agroecosystem

**DOI:** 10.3897/BDJ.9.e60745

**Published:** 2021-03-04

**Authors:** Leticia Bao, Sebastián Martínez, Mónica Cadenazzi, Mónica Urrutia, Lucía Seijas, Enrique Castiglioni

**Affiliations:** 1 Crop Protection Department, University of Republic, Montevideo, Uruguay, Montevideo, Uruguay Crop Protection Department, University of Republic, Montevideo, Uruguay Montevideo Uruguay; 2 Instituto Nacional de Investigación Agropecuaria (INIA), Programa Nacional de Investigación en Producción de Arroz, Laboratorio de Patología Vegetal, Estación Experimental INIA Treinta y Tres, Ruta 8 km 281, Treinta y Tres, C.P. 33000, Treinta y Tres, Uruguay Instituto Nacional de Investigación Agropecuaria (INIA), Programa Nacional de Investigación en Producción de Arroz, Laboratorio de Patología Vegetal, Estación Experimental INIA Treinta y Tres, Ruta 8 km 281, Treinta y Tres, C.P. 33000 Treinta y Tres Uruguay; 3 Department of Statistical Biometrics and Computing, University of Republic, Paysandú, Uruguay Department of Statistical Biometrics and Computing, University of Republic Paysandú Uruguay; 4 University of Republic, Montevideo, Uruguay University of Republic Montevideo Uruguay; 5 Eastern Region University Centre, University of Republic, Rocha, Uruguay Eastern Region University Centre, University of Republic Rocha Uruguay

**Keywords:** biodiversity, bio-indicators, water quality

## Abstract

This work is a first approach to the knowledge of insects and other aquatic macroinvertebrates of rice agroecosystems from eastern Uruguay. The composition of the groups collected may represent an approximation to the knowledge of the quality of water sources associated with Uruguayan rice production. Sampling of aquatic macroinvertebrates was carried out during the grain-filling stage in crops without insecticide use, in three localities of Treinta y Tres Department. In each crop, macroinvertebrates were collected with a Surber-type network at the inlet and outlet of water to and from the paddy field and a neighbouring control area. Differences in morphospecies composition were found according to the location and source of water. Insecta was the most represented class in macroinvertebrate samplings (41.5%). Diptera (59.9%), Hemiptera (16.3%) and Ephemeroptera (14.0%) were the most abundant orders within insects. The Richness and Shannon Diversity Indices were higher than those recorded for similar studies in Costa Rica, Italy and Australia.

## Introduction

According to the international Ramsar convention, rice fields are considered as artificial wetlands. Around 100 Ramsar sites in the world include rice areas and perform important ecological functions which sustain remarkable biodiversity (Rizo-Patrón et al. 2013). Ecosystemic services provided by rice crops include nutrient recycling, soil generation and biodiversity, with contributions that vary depending on the region (Ramsar Convention Wetlands 2018).

Natural water sources harbour more than 6% of all insect species in the world and a number of those can colonise artificial water ponds formed for rice cultivation or paddy fields (Dijkstra et al. 2014). Freshwater animal diversity is represented by approximately 126,000 species, with some underestimation of the true diversity for several important groups, which represent 10% of the total animal species actually recognised (Balian et al. 2008, Dijkstra et al. 2014). Insects represent 60% of all those known animal species reported from freshwater habitats and account for around 100,000 species, followed by vertebrates (14.5%), crustaceans (10%), arachnids (5%) and molluscs (4%) (Balian et al. 2008, Dijkstra et al. 2014).

With the reduction in natural wetlands, the occurrence of rice agroecosystems over wide areas of eastern Uruguay could substitute for different groups of animals for the loss of habitat, particularly for aquatic organisms. Therefore, in this context, rice paddies can be evaluated as habitats that can act as a viable refuge for organisms that inhabit surrounding natural habitats (Lupi et al. 2013, Wakhid et al. 2020).

The rice crop in Uruguay is produced under irrigation, so the semi-aquatic temporary environment resulting from tilling to pre-harvest could be favourable for insects or other groups that have one or more of their life stages in aquatic environments. Changes in the abundance of aquatic organisms, including insects, have been observed in relation to different rice growth phenological stages (Molozzi et al. 2007). Although marked seasonal variations in abundances of aquatic organisms have been reported, most abundance patterns in different phenological stages of rice crop have been reported as similar amongst different growing seasons (Che Salmah et al. 2017). The short period of flooding in rice fields can affect survival of some species with longer life cycles while favouring some groups (Lupi et al. 2013). When rice is flooded, the water used for irrigation is subjected to rapid chemical and physical changes, such as thermal and nutrients fluctuations. Advancing the growth stages, height and foliar area of rice plants produces thermal fluctuations that affect the creation of a microclimate in shaded areas (Lupi et al. 2013). Additionally, the abundance and diversity of aquatic organisms in rice farms can be modified under variations in food sources, habitat availability, soil drainage and management practices, mostly by machinery use and agrochemical application (Che Salmah et al. 2017). Agrochemical application is one of the main management practices affecting diversity and abundance of aquatic organisms, not only by the direct toxic effects, but also by changing the physico-chemical conditions of water (Rizo-Patrón et al. 2013, Che Salmah et al. 2017). For example, Rizo-Patrón et al. (2013) found a greater number of families and species of aquatic invertebrates in organic rice (i.e. no agrochemical use) than in conventional rice cultivation. Thus, it would also be interesting to know the impact on the conservation of the diversity of aquatic organisms in rice fields with a low input of agrochemicals (i.e. no insecticide use and limited use of fungicides), as occurs in some rice-growing areas of Uruguay (Tseng et al. 2020). This particular manner of cultivation (i.e. cultivation under irrigation during part of the crop cycle and limited use of agrochemicals) with respect to other crops and the location in wetland regions presents Uruguayan rice fields as potential biodiversity reservoirs for aquatic organisms (Maltchik et al. 2011, Luo et al. 2014). In addition, depending on the crop management, conservation of several groups of aquatic organisms could be promoted (Settle 1996, Stenert et al. 2009, Gurr et al. 2012). In the same way, natural and semi-natural habitats can provide over-wintering sites, refuges after disturbance and sources of alternative prey for natural enemies in the landscape where crop fields are located (Blitzer et al. 2012).

As different groups have different degrees of water pollution tolerance, the composition of macroinvertebrate communities would change amongst water sources and could generate important information about water quality (Rizo-Patrón et al. 2013, Melo et al. 2015).

Research related to aquatic macroinvertebrates in Uruguay refers to natural environments (Morelli and Verdi 2014), but information related to agroecosystems in the region is scarce (Rizo-Patrón et al. 2013, Vilaboa 2012, González et al. 2014), while recent research highlights the need for this information to advance the definition of environmental indicators for rice cultivation (Tseng et al. 2020). Furthermore, taking into account the extended area and the production under irrigation, the rice agroecosystems in Uruguay could have the potential to provide ecosystem services and contribute to conservation of regional wetlands biodiversity, whenever insecticide use remains in a low percentage of total productive area (Bao and Martínez 2018). Thus, obtaining information on the current state of the biodiversity of these systems is highly desirable for current decision-making in rice management tending to valuate and preserve the potential mentioned above. This does not replace the need to preserve wetland areas, since beyond the registered biodiversity, differences can be registered between the macroinvertebrate communities associated with paddy fields and wetlands given the dynamics of each of these systems (Pires et al. 2015).

This work represents the first approach to the study of aquatic organisms in rice crops and for the evaluation of the role of Uruguayan rice agroecosystems in biodiversity conservation of aquatic organisms. The information generated could help to define groups relevant for the ecosystemic services they bring and propose management measures that contribute to their conservation. In addition, group composition in different samples could represent an approximation to water quality knowledge of water sources associated with rice crop production.

## Material and methods

### Study area and sampling

Sampling was undertaken in February 2015 in three rice crops from Treinta y Tres Department in north-eastern Uruguay (Fig. 2). This Region represents the main area of rice production in Uruguay and belongs to Laguna Merín Basin, where the main production activities are rice and livestock farming. In each locality (site), each crop was sampled in three locations considering water circulation (water source), the points where water entrance (E) to the crop and water outlet (O) occurred from the crop and a control zone (C). Each C zone was a natural wetland consisting of a water body, independent from the crop water source, located at a distance of at least 50 metres from the border of the crop.

In each crop at each water source (E, O, C), three points were selected and water physicochemical properties were registered: dissolved oxygen (DO), temperature and conductivity (completely randomised design). After this, from each point, macroinvertebrates were collected with a Surber net (30 x 30 cm section) for a 1-minute period. Collected material was placed in vials containing 90% alcohol. Collected individuals were counted and organised by taxa with the aid of taxonomic keys, while insect specimens were sorted to morphospecies, the remaining macroinvertebrates were sorted to family (Springer 2006, Springer 2008, Merritt and Cummins 2007, Bentancourt et al. 2009) and functional groups were adjudicated following Ramírez and Gutiérrez-Fonseca (2014).

### Data analysis

Mean values of physicochemical properties for each water source and within each site were calculated and compared by the Tukey Test (p < 0.05). Functional groups and physicochemical properties were analysed by the Mantel Test to study the correlation between water properties and the composition of functional groups (SAS Institute 2009).

Using the insect data counting matrix, richness estimators (Chao 1 and Chao 2, Jackknife 1 and Jackknife 2, Bootstrap and ACE) were calculated and a species accumulation curve was performed with EstimateS 9.1.0 software (Colwell 2013). Species composition was analysed and compared by the ANOSIM Test through Bray-Curtis distances and the SIMPER Test was performed to determine the species that contribute to the major differences between localities and water source (PAST software, Hammer et al. 2001). Using the same software and the Bray-Curtis distances data matrix, a Principal Component Analysis (PCA) with *Biplot* was undertaken.

A heatmap was constructed with the *heatmap* function using realtive abundances of each taxonomic group (R Core Team 2020).

Abundance, Richness and Diversity Indices (Simpson, Shannon, Evenness, Margalef, Equitability and Berger-Parker) were calculated for each sample corresponding to each water source and locality (PAST Software, Hammer et al. 2001) and values were analysed through ANOVA and compared by the Tukey Test (p < 0.05) in Infostat (Di Rienzo et al. 2017).

## Results

Water physicochemical properties varied within each site and also between each water source (Table 1). Dissolved oxygen (DO) values presented statistical differences between sites (F = 5.64, p = 0.011) and between water sources (F = 4.94, p = 0.017). Conductivity values were different only between sites (F = 123.1, p = 0.0001), with higher values in La Charqueada.

**Figure 1.**
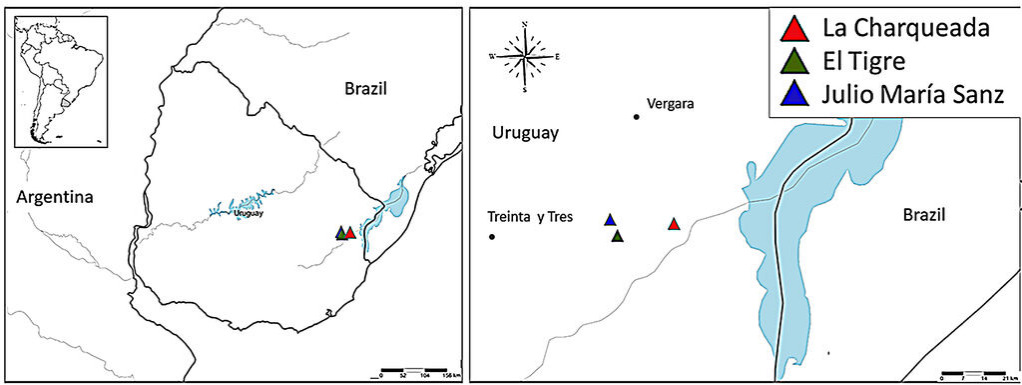
The location of sampling points for aquatic macroinvertebrates with a Surber net in the rice agroecosystem in Uruguay. Top left corner South America, with Uruguay shaded in grey, left image Uruguayan map indicating localities sampled; right, expanded image of localities sampled.

**Figure 2.**
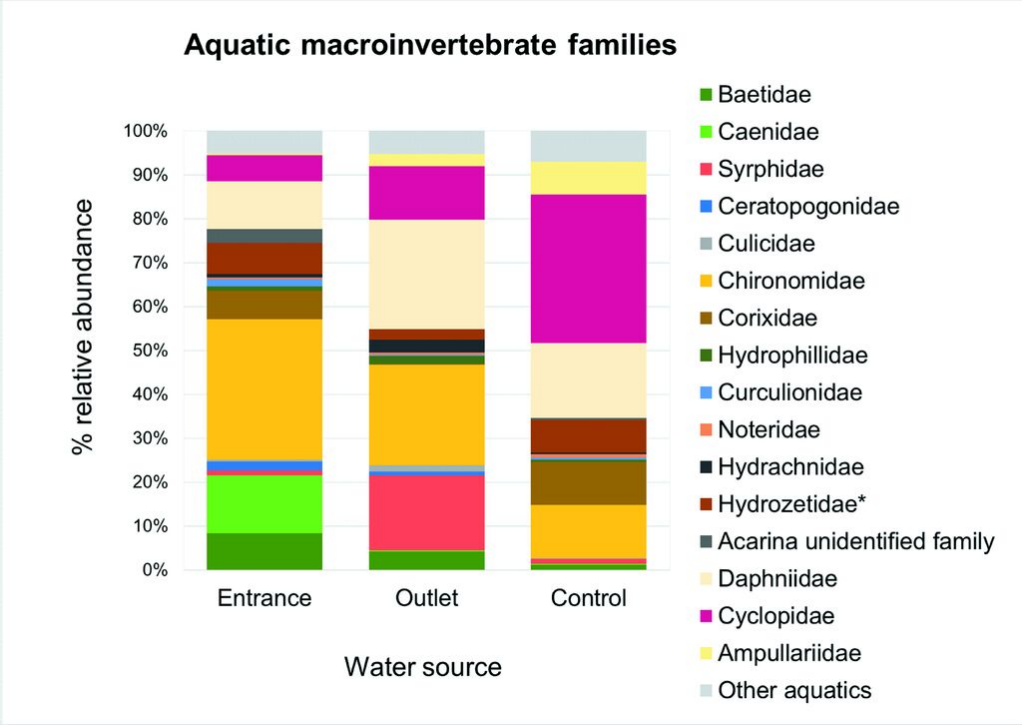
Relative abundances of aquatic macroinvertebrates collected with a Surber net in the rice agroecosystem, grouped by water source. *indicates uncertain identification at this level.

A total of 2820 macroinvertebrates in 27 samples were collected from the three localities. Insects represented 41.5% of total macroinvertebrates collected and Maxillopoda (Crustacea), Branchiopoda (Crustacea) and Arachnida were the most abundant macroinvertebrates classes collected after insects (Table 2, Fig. 1).

From the total macroinvertebrates collected, 1170 were insects, belonging to 10 orders, 39 families and 50 species/morphospecies (Table 3). The more abundant insect orders were Diptera (59.9%), Hemiptera (16.3%) and Ephemeroptera (14.0%). Chironomidae and Syrphidae were the main dipteran families with 43.9% and 13.8% of the total insect abundance, respectively. Corixidae was the most abundant hemipteran (15.0%), while Ephemeroptera was represented by Baetidae and Caenidae with 7.8% and 6.2% of the total insect abundance, respectively. The more diverse insect groups were Coleoptera with 12 families and 16 morphospecies and Diptera with seven families and 10 morphospecies.

**Figure 3.**
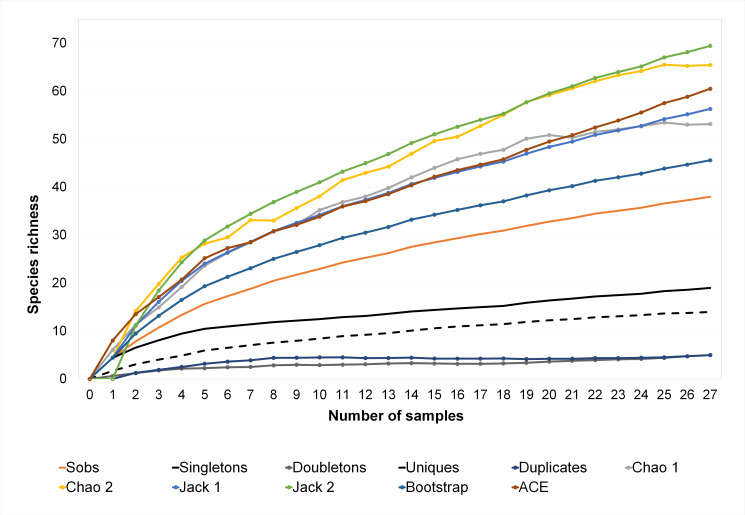
Cumulative Species Curve for aquatic insects, indicating observed richness (*Sobs*), *singletons*, *doubletons*, *duplicates*, *uniques* and richness estimators (500 aleatorisations).

The Cumulative Species Curve for insects shows that there are still species pending collection. Richness estimators show that at least 58.8% of expected species were sampled (Jackknife 2: 58.8%, Chao 1: 59.8%, Chao 2: 60.4%, ACE: 62.1%, Jackknife 1: 66.4%, Bootstrap: 74.4%, corresponding to an estimated number of species of 49, 48, 48, 47, 44 and 39, respectively) (Fig. 3).

The physicochemical properties of water were correlated with functional groups according to the Mantel Test through Euclidean distances (r = 0.48 p = 0.019), but due to the number of samples, it was not possible to correlate each property to a particular functional group.

The Kruskal-Wallis Test for total arthropods number showed the higher values from the water outlet and control zone, with a lower count for the water entrance (H = 18.47, p = 0.0178). Following the same analysis and comparing at family level, there were also differences found between water sources for Syrphidae larvae, with higher abundances in the water outlet (H = 20.34, p = 0.006) (Table 4).

**Figure 4.**
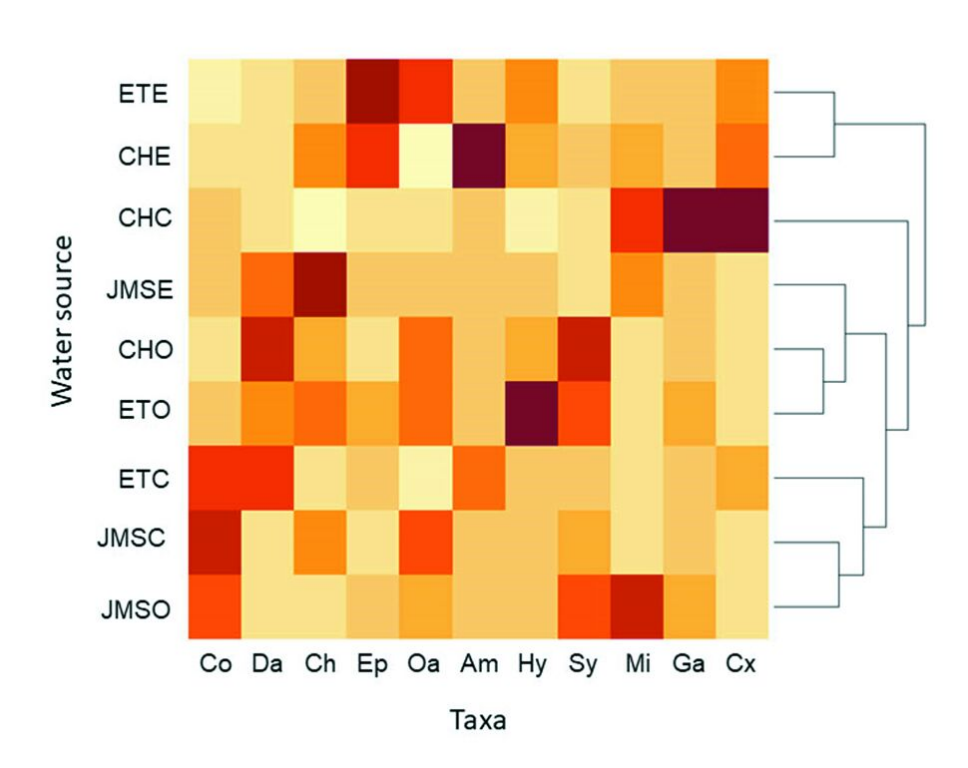
Heat map of taxon relative abundance and hierarchical clustering of samples. References: Sites: CH: La Charqueada, JMS: Julio María Sanz, ET: El Tigre; Water source: E: entrance, O: outlet, C: control; Taxonomic group: Co: copepods, Da: Daphnia, Ch: Chironomidae, Ep: Ephemeroptera, Oa: Other aquatics, Am: Amphipoda, Hy: Hydrophilidae, Sy: Syrphidae, Mi: Mites, Ga: Gastropoda, Cx: Corixidae. Values are scaled by taxon relative abundances across each site and water source.

Taxa distributions by site and water source are shown as a heat map (Fig. 4). Despite all groups being registered in all the environments, some taxa were more abundant in certain water sources or sites. For example, Ephemeroptera were more abundant at the water entrances, while Syrphidae were more abundant in water outlet samples and Corixidae were more abundant in the control zones.

The ANOSIM Test showed differences according to locality and water source (R = 0.388, p = 0.0013 and R = 0.615, p = 0.0001, respectively). According to the SIMPER Test through Bray-Curtis distances, 80% of the differences between localities was explained by seven species, while differences between water sources were explained by six species (Table 5).

**Figure 5.**
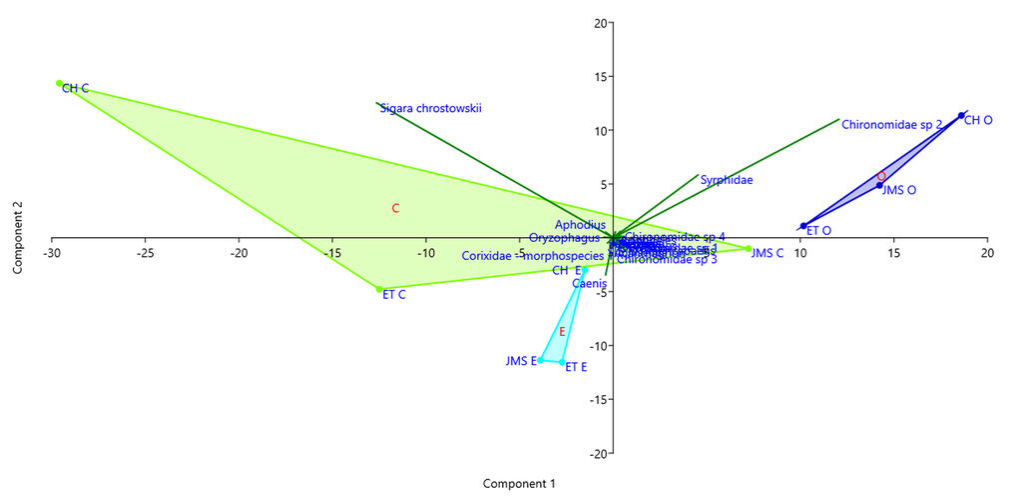
PCA test with *Biplot* from variance-covariance matrix of Bray-Curtis distances for each locality and water source. (Control: C in green. Entrance: E in sky-blue and Outlet: O in blue). Component 1: 57.74% and Component 2: 21.50% of variance, respectively.

The Principal Component Analysis (PCA) shows samples grouping according to the water source (Fig. 5). Chironomidae morphospecies 2 and Syrphidae (Diptera) were associated with water outlet samples, while *Caenis* (Ephemeroptera) was associated with water entrance samples and corixids (*Sigara chrostowskii*) to the water control zone.

Richness and Diversity Indices are shown in Table 6. Values for control zones did not show differences between sites, while, in the case of the water inlet, higher values of agreement were registered for El Tigre. Water outlet indices showed differences in some values, with the highest specific diversity in El Tigre (mean of 13 species).

Comparing values within each field according to water source, the major differences were registered for El Tigre. In J.M. Sanz, differences were registered only for richness with the higher value at the water entrance. On the other hand, for Charqueada, no differences were registered between water sources.

## Discussion

This study represents the first description of the aquatic macroinvertebrate community associated with rice crops in Uruguay and establishes a baseline for present and future studies on productivity and ecological sustainability of Uruguayan rice farms.

In relation to water physicochemical properties, the greatest differences were registered for dissolved oxygen with lower values in control zones from El Tigre and J.M. Sanz. Control zones are water bodies located in crop surroundings and with less area and without water flux compared with rice areas. In the case of La Charqueada, the control zone belongs to a more extensive water body. Less oxygen content in El Tigre and J. M. Sanz could be due to water body characteristics of these localities compared to La Charqueada control zone, such as the size and sun radiation or vegetable coverage, for example (Ito 1987).

The Mantel Test demonstrated an effect of physicochemical properties on the guild composition in different water sources. Richness estimators show that there are still many species remaining to be collected and this work recorded at least 58% of the species, depending on the estimator considered. However, even under these conditions, from the 50 morphospecies collected, 36 were aquatic insects, showing a greater diversity than reported previously in tropical-subtropical rice fields of Brazil (34 morphospecies, Molozzi et al. 2007), but less diversity than that found in Costa Rica (44 insect morphospecies, Rizo-Patrón et al. 2013) and Indonesia (45 insect morphospecies, Wakhid et al. 2020). However, our data are the results of sampling only at the grain-filling stage, thus biodiversity could be underestimated. It is important to emphasise that Uruguayan rice crops are mainly managed in a rotation system with pastures, which could bring more stability to the agroecosystem and sustain greater diversity (Norton et al. 2010). In addition, this production system has low inputs of insecticides which could also contribute to retaining biodiversity and natural pest control (Ali et al. 2019).

Insecta was the most represented Class in the samples, with Diptera, Hemiptera and Ephemeroptera as the most represented Orders. The most abundant aquatic insects found in the present work were Chironomidae (Diptera), the most ubiquitous family of aquatic flies with a worldwide distribution (Ferrington 2008, Dijkstra et al. 2014). Chironomids include species which are detritivores and can function as alternative food sources for generalist predators like Arachnids and Odonata (Ferrington 2008; Wakhid et al. 2020). Moreover, further work on chironomid species identification would allow them to be assessed for water quality monitoring (Takahashi et al. 2008).

The ANOSIM Test proved that there were differences in morphospecies composition according to locality and water source (R = 0.388, p = 0.0013 and R = 0.615, p = 0.0001, respectively). Some groups showed association with a particular water source, so they could be analysed for their potential as water quality indicators for each source studied (Entrance, Outlet and Control zone). *Caenis* (Ephemeroptera) individuals are associated with the water entrance. It is well documented that *Caenis* individuals are sensitive to pollution (Roldán 2003, Ocón and Rodríguez-Capítulo 2012), so the greater amounts observed in water entrances could be indicating better water quality compared to outlet and control zones. On the contrary, a higher abundance of Syrphidae larvae in water outlets could be indicating water deterioration in the path through the crop or soil and plant debris addition from the rice crop due to these organisms being associated with an excess of organic matter (Bonada et al. 2006, Arimoro et al. 2007). However, in control zones, corixids were more abundant and the species varied between sampling localities. Corixids are classified as predators and several species are pioneers that quickly colonise new habitats, occasionally newly-planted rice fields (Wakhid et al. 2020).

Coleoptera was the most diverse order (18 morphospecies) and Dytiscidae was the most diverse family (four morphospecies). These results are in accordance with data reported by Gómez Lutz et al. (2015) for Argentinean rice fields that reported Dytiscidae and Hydrophilidae as the most diverse coleopteran families. Taking into account these results, further sampling exercises could probably register an even higher number of coleopteran species for Uruguayan rice fields.

The Shannon-Weaver Indices, calculated for locality and water source with aquatic insects species, did not show differences between water sources. However, richness values registered for aquatic insect species are higher than those registered in organic rice crops in Australia (Wilson et al. 2008), but lower than those of Costa Rica and Italy (Rizo-Patrón et al. 2013, [Bibr B6310790][Bibr B6311122]). Considering richness estimators calculated in this work and the fact that sampling was only during the grain-filling stage, we expect to find more species with future research. In relation to similar research from Uruguay, Shannon Indices are similar to those reported by Morelli and Verdi (2014) in watercourses from riverine native vegetation, despite the fact that species composition was different.

The higher richness and diversity registered in El Tigre could be due to different factors. From the point of view of the landscape where this crop is located, probably there is a better connectivity with native vegetation patches than in the other two sites (J.M. Sanz and La Charqueada), but this has to be confirmed. It is possible that this environment, with the largest area of native riverine vegetation of the three sampled sites, could be a better reservoir for species, supporting a higher diversity and richness (Molozzi et al. 2007, González et al. 2016).

Moreover, it should be emphasised that sampling was undertaken at the grain-filling stage, so there is no information about possible diversity fluctuation along the crop cycle, as reported previously (Che Salmah et al. 2017). Under these sampling conditions, it is important to discuss the fact that diversity could have been underestimated and there could be still many macroinvertebrate species associated with this crop that are pending to be sampled. A more exhaustive sampling at different crop stages could detect more differences in macroinvertebrate community composition and register an even higher number of species for Uruguayan rice ecosystems.

The progress in aquatic macroinvertebrate community characterisation and agrochemical residues evaluations could enable the definition of indicators as relatively quick sampling tools for water quality screening, thus enabling the measurement of the impact of crop management activities.

## Conclusions

The physicochemical properties of water had an effect on the composition of the guilds. Different morphospecies compositions were found according to locality and water source. *Caenis* individuals were associated with the water entrance, while Syrphidae and Chironomidae (morphospecies 2) larvae were associated with the water outlet. Some of these species could be useful indicators of water quality. Arthropods were more abundant in the water of control zones. More exhaustive sampling could register more diversity and potential bio-indicators. Richness and diversity indices registered in this work - even with an inventory of species pending registration - are higher than those reported for rice agroecosystems from Costa Rica, Italy and Australia.

## Figures and Tables

**Table 1.** Physicochemical properties of water at different localities and in different water sources (entrance: E, outlet: O and Control zone: C) in rice agroecosystem in Treinta y Tres, Uruguay.

**Locality (site)**	**Water source**	**Temperature (°C)**	**DO (O_2_ mg/l)**	**Conductivity (µS)**
La Charqueada (33°11.824'S 53°50.301'W)	Entrance	26.57 ±0.64a	4.20±0.89abc	163.67±10.02ab
	Outlet	26.47±0.64a	6.27±0.89c	139.30±10.02a
	Control	30.10±0.64b	6.16±0.89c	180.50±12.27b
El Tigre (33°13.625'S 54°0.387'W)	Entrance	27.53±0.57a	9.71±0.89b	89.73±3.79a
	Outlet	29.63±0.57b	9.63±0.89b	94.73±3.79a
	Control	28.23±0.57ab	1.95±0.89a	102.63±3.79a
Julio M. Sanz (33°11.201'S 54°1.698'W)	Entrance	26.50±0.11a	2.67±0.89ab	59.93±2.03a
	Outlet	27.40±0.11b	5.06±0.89bc	60.95±2.03a
	Control	27.30±0.11b	2.21±0.89a	58.00±2.48a

**Table 2.** Relative abundance of macroinvertebrates (no insects) collected with a Surber net in the rice agroecosystem, in Treinta y Tres, Uruguay.

**Class**	**Order**	**Family**	**Genus/morpho species**	**Relative abundance (%)**
Maxillopoda	Cyclopoida	Cyclopidae	Cyclopidae morphospecies 1	22.50
Branchiopoda	Anomopoda	Daphniidae	*Daphnia* morphospecies 1	18.14
Arachnida	Trombidiformes (subclass Acarina)	Hydrachnidae Hydrozetidae^ undetermined	*Hydrachna* morphospecies 1 Hydrozetidae morphospecies 1 Acarina morphospecies 1	8.00
Gastropoda (snails)	Caenogastropoda	Ampullariidae	*Pomacea* and other spp.	4.88
Oligochaeta	Tubificida	undetermined	Tubificida morphospecies 1	1.80
Malacostraca	Amphipoda	Hyalellidae	*Hyalella* morphospecies 1^	1.39
Arachnida	Araneae	Allocosinae, Lyniphiidae, Anyphaenidae	juveniles	0.33
Clitellata	Hirudinea	undetermined	unidentified leech	0.29

^ indicates uncertain identification at Genus level.

**Table 3.** Insects collected with a Surber net in the rice agroecosystem at the entrance, outlet and control zones of water in Treinta y Tres. Uruguay.

**Order**	**Family**	**Genus/Species/morphospecies**
Ephemeroptera	Baetidae	*Americabaetis* morphospecies 1^
	Caenidae	*Caenis* morphospecies 1
Odonata	Libellulidae	Libellulidae morphospecies 1
	Coenagrionidae	*Acanthagrion* morphospecies 1 ^
Trichoptera	Hydroptylidae	*Oxyethira* morphospecies 1
Hemiptera	Belostomatidae	*Belostoma* morphospecies 1
	Corixidae	*Sigara chrostowskii*
		Corixidae morphospecies 1
	Notonectidae	Notonectidae morphospecies 1
	Nepidae	*Ranatra* morphospecies 1
	Aphididae	Rhopalosiphum padi*
	Cicadellidae	Cicadellidae morphospecies 1 (nymph)*
	Pentatomidae	Pentatomidae morphospecies 1 (nymph)*
	Lygaeidae	Nysius simulans*
	Cercopidae	Cercopidae morphospecies 1 (nymph)*
Hymenoptera	Formicidae	*Solenopsis* morphospecies 1*
Coleoptera	Hydrophylidae	*Berosus* morphospecies 1
		*Tropisternus* morphospecies 1
	Dytiscidae	*Enochrus* morphospecies 1
		*Laccophillus* morphospecies 1
		*Rhantus* morphospecies 1
		*Desmopachria* morphospecies 1^
	Curculionidae	*Oryzophagus oryzae*
		*Hypselus ater*
	Scarabaeidae	*Ataenius* morphospecies 1
		*Aphodius* morphospecies 1
	Haliplidae	*Haliplus* morphospecies 1
	Carabidae	*Meotachys* morphospecies 1^*
	Elmidae	Elmidae morphospecies 1
	Ptinidae	*Scydmaenus* morphospecies 1 *
	Chrysomelidae	*Epitrix* morphospecies 1*
	Mycetophagidae	Mycetophagidae morphospecies 1*
	Staphylinidae	Staphylinidae morphospecies 1*
	Noteridae	*Suphisellus* morphospecies 1
Diptera	Chironomidae	Chironomidae morphospecies 1
		Chironomidae morphospecies 2
		Chironomidae morphospecies 3
		Chironomidae morphospecies 4
		Chironomidae morphospecies 5
	Syrphidae	Eristalinae morphospecies 1
	Ceratopogonidae	*Bezzia* morphospecies 1
	Culicidae	*Anopheles* morphospecies 1
	Simuliidae	Simuliidae morphospecies 1
	Empididae	Empididae morphospecies 1
Lepidoptera	Piralidae	*Parapoynx* morphospecies 1
	Hesperidae	*Hylephila* morphospecies 1
Orthoptera	Tettigoniidae	*Conocephalus* morphospecies 1*
	Acridoidea	subfamily Acridinae morphospecies 1*
Thysanoptera	Thripidae	Thripidae morphospecies 1*
Psocoptera		Psocoptera morphospecies 1*

* terrestrial groups not included in analysis.

^ indicates uncertain identification at this level.

**Table 4.** Abundance of Syrphidae larvae and total number of arthropods according to water source (Entrance, Outlet and Control) in the rice agroecosystem in Treinta y Tres. Uruguay.

**Water source**	**Syrphidae larvae**	**Total number of arthropods**
Entrance	0.67 ± 1.32a	40.78 ± 6.87a
Outlet	15.56 ± 11.54b	89.11 ± 16.61b
Control	1.22 ± 1.30a	145.78 ± 18.56c

The same letter within each column means no significant differences LSD Fisher Test (p ˃ 0.05).

**Table 5.** SIMPER Test with species accomplishing 80% dissimilarity between localities and between water circulation in the rice agroecosystem in Treinta y Tres, Uruguay.

**SIMPER Test by LOCALITY**						
Taxon	Dissimilarity	% Contribution	% Cumulative	Mean by site		
				La Charqueada	JM Sanz	El Tigre
Chironomidae morphospecies 2	22.83	27.94	27.94	13.60	15.20	7.67
*Sigara chrostowskii*	13.64	16.70	44.64	14.80	0.00	3.67
Eristalinae morphospecies 1	8.08	9.88	54.52	7.89	2.11	1.89
*Caenis* morphospecies 1	6.47	7.92	62.44	5.33	0.11	2.22
*Americabaetis* morphospecies 1 ^	6.12	7.50	69.93	3.11	1.11	4.33
Chironomidae morphospecies 3	6.07	7.43	77.36	1.89	3.11	1.00
*Oryzophagus oryzae*	2.02	2.47	79.83	0.56	0.22	0.67
**SIMPER Test by WATER SOURCE**						
Taxon	Dissimilarity	% Contribution	% Cumulative	Mean by water source		
				Entrance	Outlet	Control
Chironomidae morphospecies 2	26.06	31.02	31.02	5.11	25.10	6.22
*Sigara chrostowskii*	13.30	15.83	46.85	2.56	0.11	15.80
Eristalinae morphospecies 1	8.34	9.93	56.79	1.78	9.11	1.00
*Caenis* morphospecies 1	6.76	8.05	64.84	7.56	0.11	0.00
*Americabaetis* morphospecies 1^	6.40	7.62	72.46	4.11	4.11	0.33
Chironomidae morphospecies 3	6.26	7.45	79.91	3.33	2.33	0.33

^ indicates uncertain identification at Genus level.

**Table 6.** Richness and Diversity Indices for insects ± E.E, by locality (site) in water samples.

**Diversity**	El Tigre	J.M. Sanz	La Charqueada
	Entrance		
Richness (S)	6.33 ± 1.53aA	9.00 ± 1.73aB	7.67 ± 1.53aA
Shannon H	1.55 ± 0.12aA	1.54 ± 0.12aA	1.64 ± 0.12aA
Simpson 1-D	0.76 ± 0.03aA	0.74 ± 0.03aB	0.76 ± 0.03aA
Equitability J	0.81 ± 0.03abA	0.85 ± 0.03bB	0.700 ± 0.03aA
	Outlet		
Richness (S)	13.33 ± 0.58bB	4.67 ± 1.15aA	5.33 ± 1.53aA
Shannon H	2.02 ± 0.07bB	1.14 ± 0.07aA	1.14 ± 0.07aA
Simpson 1-D	0.62 ± 0.04aA	0.81 ± 0.04bB	0.61 ± 0.04aA
Equitability J	0.70 ± 0.07aA	0.78 ± 0.07aB	0.77 ± 0.07aA
	Control		
Richness (S)	11.00 ± 2.65aB	7.00 ± 1.73aAB	7.33 ± 2.31aA
Shannon H	1.40 ± 0.15aA	1.19 ± 0.15aA	1.05 ± 0.15aA
Simpson 1-D	0.48 ± 0.08aA	0.62 ± 0.08aA	0.58 ± 0.08aA
Equitability J	0.56 ± 0.09aA	0.59 ± 0.09aA	0.62 ± 0.09aA

The same capital letter within each column and same lower-case letter within each row means no significant differences in LSD Fisher Test (p ˃ 0.05).
